# Walking for Transportation or Leisure Among U.S. Women and Men — National Health Interview Survey, 2005–2015

**DOI:** 10.15585/mmwr.mm6625a1

**Published:** 2017-06-30

**Authors:** Emily N. Ussery, Susan A. Carlson, Geoffrey P. Whitfield, Kathleen B. Watson, David Berrigan, Janet E. Fulton

**Affiliations:** ^1^Epidemic Intelligence Service, CDC; ^2^Division of Nutrition, Physical Activity, and Obesity, National Center for Chronic Disease Prevention and Health Promotion, CDC; ^3^Division of Cancer Control & Population Sciences, National Cancer Institute, Bethesda, Maryland.

Physical activity confers considerable health benefits, but only half of U.S. adults report participating in levels of aerobic physical activity consistent with guidelines (*1*,*2*). *Step It Up! The Surgeon General’s Call to Action to Promote Walking and Walkable Communities* identified walking as an important public health strategy to increase physical activity levels (*3*). A previous report showed that the self-reported prevalence of walking for transportation or leisure increased by 6 percentage points from 2005 to 2010 (*4*), but it is unknown whether this increase has been sustained. CDC analyzed National Health Interview Survey (NHIS) data from 2005 (26,551 respondents), 2010 (23,313), and 2015 (28,877) to evaluate trends in the age-adjusted prevalence of self-reported walking among adults aged ≥18 years. The prevalence of walking increased steadily among women, from 57.3% in 2005, to 62.5% in 2010, and to 65.1% in 2015 (significant linear trend). Among men, a significant linear increase in reported walking was observed, from 54.3% in 2005, to 61.8% in 2010, and to 62.8% in 2015, although the increase stalled between 2010 and 2015 (significant linear and quadratic trends). Community design policies and practices that encourage pedestrian activity and programs tailored to the needs of specific population subgroups remain important strategies for promoting walking (*3*).

NHIS is a continuous in-person survey of U.S. households designed to be representative of the civilian, noninstitutionalized population (*5*). NHIS consists of a core questionnaire that collects basic health and demographic information for all family members in a sampled household and supplements that collect information about specialized topics. Questions specific to walking for leisure and transportation were asked of one adult aged ≥18 years per sampled household in the 2005, 2010, and 2015 Cancer Control Supplements. Sample adult response rates were 69.0% (2005), 60.8% (2010), and 55.2% (2015) (*6*).

Walking was defined as engaging in at least one 10-minute period of transportation or leisure walking in the past 7 days at the time of survey. To assess transportation walking, respondents in all 3 years were asked, “During the past 7 days, did you walk to get someplace that took you at least 10 minutes?” To assess leisure-time walking, respondents in 2005 were asked, “During the past 7 days, did you walk for at least 10 minutes at a time [for fun, relaxation, exercise, or to walk the dog]?” and in 2010 and 2015, “During the past 7 days, did you walk for at least 10 minutes [for fun, relaxation, exercise, or to walk the dog]?”

Demographic characteristics (sex, age, race/ethnicity, and education level) and health-related characteristics (height, weight, walking assistance status, and physical activity) were also assessed. Meeting the aerobic physical activity guideline of at least 150 minutes of moderate-intensity equivalent aerobic activity per week was assessed using responses on the usual frequency and duration of light- to moderate-intensity and vigorous-intensity leisure-time physical activity (*1*).

From the initial total sample of 92,257 (31,428 [2005]; 27,157 [2010]; and 33,672 [2015]), 13,516 (15%) persons were excluded, including 2,280 who were unable to walk and 11,236 for whom data were missing for walking (6,044), physical activity (1,054), health-related characteristics (3,708), or demographic characteristics (430). Thus, the final analytic sample consisted of 78,741 respondents (26,551 [2005]; 23,313 [2010]; and 28,877 [2015]).

The proportion (with 95% confidence intervals) of adults who reported walking each year was calculated. Linear and quadratic trends in walking prevalence from 2005 to 2015 were tested using logistic regression, controlling for age group. For three time points, a temporal change that includes significant linear and quadratic trend terms indicates an overall increase or decrease over time as well as a deviation from linearity. For example, if the linear trend is positive and quadratic trend is negative, this indicates an increase from 2005 to 2015 with a stalling or leveling off between 2010 and 2015. Because significant interactions between sex and trend terms were observed, sex-specific results are presented. Subgroup analyses were conducted by age group, race/ethnicity, education level, U.S. Census region, body mass index category, walking assistance status, and meeting the aerobic physical activity guideline, and pairwise differences between subgroups and across years were tested using adjusted Wald tests. Statistically significant (p<0.05) results are reported. All analyses accounted for the complex survey design. Reported estimates are weighted and age-standardized to the 2000 U.S. standard population (*7*).

In 2015, women were significantly more likely to report walking (65.1%) than were men (62.8%) (Figure). Among women in 2015, the lowest reported prevalence of walking was among those aged ≥65 years, non-Hispanic blacks (blacks), and residents of the South, compared with their respective counterparts (Table 1). Among men in 2015, the lowest prevalence of walking was among blacks and Hispanics and the highest prevalence was among men in the West, compared with their respective counterparts (Table 2). Among males, there were no significant age group differences in walking prevalence. The prevalence of walking was lower among men and women with a high school education or less, who had obesity, who needed walking assistance, or who did not meet aerobic physical activity guidelines than among their respective counterparts.

**FIGURE:**
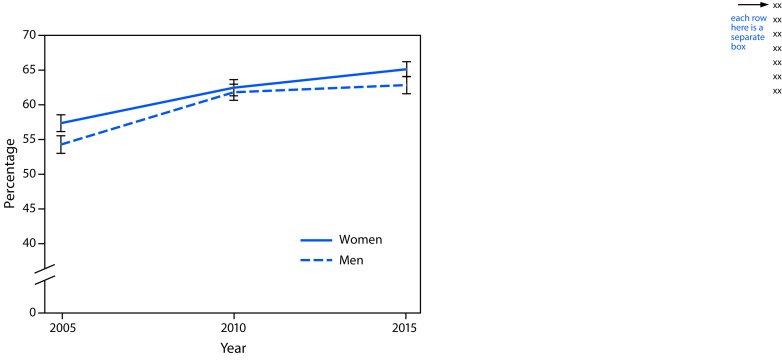
Percentage* of U.S. women^†^ and men^§^ aged ≥18 years who reported recent walking for transportation or leisure — National Health Interview Survey, 2005–2015 * Weighted percentages, age-standardized to the 2000 U.S. standard population. Error bars represent upper and lower bounds of 95% confidence intervals. ^†^ Significant linear trend from 2005 to 2015 only (p<0.05), based on trend analyses using logistic regression controlling for age category. ^§^ Significant linear trend from 2005 to 2015 (p<0.05) and a significant deviation from linear trend (p<0.05), based on trend analyses using logistic regression controlling for age category.

**TABLE 1:** Proportion of U.S. women aged ≥18 years who reported recent walking for transportation or leisure, by selected demographic and health characteristics — National Health Interview Survey, 2005–2015

Characteristic	%* (95% CI)			Absolute change from 2010 to 2015
	2005	2010	2015	
	(n = 14,609)	(n = 12,734)	(n = 15,562)	
**Total**	57.4 (56.1–58.6)	62.5 (61.3–63.6)	65.1 (64.0–66.2)^†^	2.7^§^
**Age group (yrs)**				
18–24	61.4 (58.0–64.7)	65.4 (62.1–68.6)	66.2 (62.5–69.8)	0.8
25–34	59.7 (57.3–62.1)	66.6 (64.1–69.0)	69.0 (66.7–71.2)^†^	2.4
35–44	62.1 (59.9–64.3)	66.2 (63.8–68.5)	68.4 (65.9–71.0)^†^	2.2
45–64	56.7 (54.9–58.6)	62.8 (61.0–64.6)	65.7 (63.8–67.5)^†^	2.9^§^
≥65	46.8 (44.6–49.0)	50.6 (48.1–53.0)	55.0 (52.8–57.2)^†^	4.4^§^
**Race/Ethnicity**				
White, non-Hispanic	59.5 (58.0–60.9)	64.0 (62.6–65.5)	66.6 (65.2–68.1)^†^	2.6^§^
Black, non-Hispanic	47.5 (45.0–50.1)	53.8 (51.2–56.5)	55.5 (52.4–58.5)^†^	1.7
Hispanic	54.0 (51.0–57.0)	60.6 (58.2–63.0)	63.9 (61.5–66.3)^†^	3.3
Other race^¶^	59.2 (55.2–63.3)	66.9 (63.8–69.9)	69.9 (66.6–73.3)^†^	3.0
**Education level**				
Less than high school graduate	47.0 (44.3–49.7)	51.2 (48.4–54.0)	55.1 (52.2–58.0)^†^	3.9
High school graduate	49.8 (47.8–51.9)	55.6 (53.4–57.9)	56.4 (54.1–58.7)^†^	0.8
Some college	59.9 (57.9–61.8)	63.4 (61.3–65.4)	63.7 (61.9–65.6)^†^	0.3
College graduate	68.5 (66.3–70.7)	72.4 (70.3–74.4)	76.0 (74.2–77.8)^†^	3.6^§^
**U.S. Census region**				
Northeast	66.1 (63.8–68.5)	65.7 (62.8–68.5)	70.4 (68.0–72.8)^†^	4.7^§^
Midwest	56.7 (54.3–59.0)	62.6 (60.4–64.9)	62.9 (60.8–65.1)^†,^**	0.3
South	50.8 (48.6–52.9)	56.4 (54.3–58.4)	59.9 (57.9–61.9)^†^	3.6^§^
West	61.8 (59.4–64.2)	69.2 (66.9–71.5)	71.8 (69.8–73.8)^†^	2.6
**Body mass index category^††^**				
Underweight/Normal weight	61.4 (59.9–62.9)	66.6 (65.0–68.2)	70.3 (68.8–71.8)^†^	3.7^§^
Overweight	56.7 (54.6–58.7)	63.8 (62.0–65.6)	65.0 (63.1–66.9)^†,^**	1.2
Has obesity	50.0 (47.9–52.0)	54.6 (52.5–56.8)	57.8 (55.9–59.7)^†^	3.1^§^
**Walking assistance status^§§^**				
Does not need assistance	59.7 (58.5–61.0)	65.3 (62.6–64.9)	67.9 (63.1–65.6)^†^	2.6^§^
Needs assistance	25.8 (20.5–31.0)	23.6 (19.3–34.2)	30.3 (23.1–35.6)	6.7
**Meets aerobic physical activity guideline^¶¶^**				
No	44.6 (43.2–46.1)	49.1 (47.6–50.7)	51.0 (49.5–52.6)^†^	1.9
Yes	76.8 (75.4–78.1)	79.3 (78.0–80.7)	80.6 (79.3–82.0)^†^	1.3

**Abbreviation:** CI = confidence interval.

* Weighted percentages, age-standardized to the 2000 U.S. standard population.

^†^ Significant linear trend from 2005 to 2015 (p<0.05), based on trend analyses using logistic regression controlling for age category.

^§^ Significant change from 2010 to 2015 (p<0.05).

^¶^ “Other race” category includes non-Hispanic Asian, non-Hispanic American Indian/Alaskan Native, and persons reporting more than one race.

** Significant deviation from linear trend from 2005 to 2015 (p<0.05), based on trend analyses using logistic regression controlling for age category.

^††^ Body mass index (weight [kg]/height [m^2^]) estimates were calculated from self-reported weight and height. Underweight and normal weight: <25.0, overweight: 25.0–29.9, and has obesity: ≥30.

^§§^ Needing walking assistance was defined as being unable or finding it very difficult “to walk one-quarter mile without special equipment.”

^¶¶^ Meeting the 2008 aerobic physical activity guideline was defined as participating in ≥150 minutes of moderate-intensity equivalent aerobic activity per week (light- to moderate-intensity minutes plus two times vigorous-intensity minutes).

**TABLE 2:** Proportion of U.S. men aged ≥18 years who reported recent walking for transportation or leisure, by selected demographic and health characteristics — National Health Interview Survey, 2005–2015

Characteristic	%* (95% CI)			Absolute change from 2010 to 2015
	2005	2010	2015	
	(n = 11,942)	(n = 10,579)	(n = 13,315)	
**Total**	54.3 (53.0–55.5)	61.8 (60.6–63.0)	62.8 (61.6–64.1)^†,§^	1.0
**Age group (yrs)**				
18–24	56.0 (52.5–59.4)	65.7 (62.2–69.3)	63.6 (59.8–67.5)^†,§^	−2.1
25–34	52.5 (50.0–55.0)	63.7 (61.0–66.3)	64.5 (61.9–67.2)^†,§^	0.8
35–44	54.4 (52.1–56.7)	61.3 (58.7–63.9)	62.3 (59.2–65.3)^†^	1.0
45–64	54.5 (52.7–56.4)	61.8 (60.0–63.7)	62.8 (60.8–64.8)^†,§^	1.0
≥65	54.3 (51.6–56.9)	57.4 (54.6–60.2)	61.2 (58.9–63.5)^†^	3.8^¶^
**Race/Ethnicity**				
White, non-Hispanic	55.1 (53.6–56.6)	62.9 (61.5–64.3)	64.1 (62.4–65.8)^†,§^	1.2
Black, non-Hispanic	50.8 (47.9–53.7)	55.5 (52.3–58.7)	58.3 (55.2–61.4)^†^	2.8
Hispanic	52.5 (49.6–55.3)	60.1 (57.4–62.8)	59.6 (56.7–62.5)^†,§^	−0.5
Other race**	53.8 (48.5–59.1)	64.5 (60.6–68.4)	67.6 (64.2–71.1)^†^	3.1
**Education level**				
Less than high school graduate	46.1 (43.6–48.7)	53.8 (51.1–56.5)	53.3 (50.0–56.6)^†,§^	−0.5
High school graduate	46.5 (44.4–48.5)	55.5 (53.3–57.6)	56.2 (53.7–58.6)^†,§^	0.7
Some college	55.7 (53.7–57.8)	61.6 (59.5–63.7)	61.0 (58.8–63.2)^†,§^	−0.6
College graduate	64.8 (62.4–67.2)	71.5 (69.3–73.7)	72.8 (70.8–74.9)^†^	1.3
**U.S. Census region**				
Northeast	61.8 (58.9–64.6)	66.2 (63.5–69.0)	63.7 (60.8–66.6)^§^	−2.6
Midwest	54.2 (51.7–56.6)	60.4 (58.0–62.7)	61.0 (58.5–63.5)^†,§^	0.6
South	47.8 (45.7–50.0)	57.5 (55.4–59.6)	59.6 (57.5–61.7)^†,§^	2.2
West	58.8 (56.0–61.6)	66.3 (64.0–68.7)	68.7 (66.0–71.5)^†^	2.4
**Body mass index category^††^**				
Underweight/Normal weight	54.8 (52.7–56.9)	63.9 (62.0–65.9)	64.3 (62.0–66.5)^†,§^	0.4
Overweight	55.8 (54.0–57.6)	62.8 (61.0–64.6)	63.0 (61.3–64.8)^†,§^	0.2
Has obesity	51.8 (49.5–54.1)	58.2 (56.1–60.4)	60.8 (58.4–63.2)^†^	2.6
**Walking assistance status^§§^**				
Does not need assistance	55.8 (54.5–57.1)	63.8 (62.6–64.9)	64.4 (63.1–65.6)^†,§^	0.6
Needs assistance	26.6 (19.5–33.8)	26.7 (19.3–34.2)	29.3 (23.1–35.6)^†^	2.6
**Meets aerobic physical activity guideline^¶¶^**				
No	41.0 (39.3–42.6)	48.4 (46.7–50.1)	47.5 (45.5–49.4)^†,§^	−0.9
Yes	70.5 (69.0–71.9)	74.5 (73.1–76.0)	76.2 (74.8–77.5)^†^	1.7

**Abbreviation:** CI = confidence interval.

* Weighted percentages, age-standardized to the 2000 U.S. standard population.

^†^ Significant linear trend from 2005 to 2015 (p<0.05), based on trend analyses using logistic regression controlling for age category.

^§ ^Significant deviation from linear trend from 2005 to 2015 (p<0.05), based on trend analyses using logistic regression controlling for age category.

^¶^ Significant change from 2010 to 2015 (p<0.05).

** “Other race” category includes non-Hispanic Asian, non-Hispanic American Indian/Alaskan Native, and persons reporting more than one race.

^††^ Body mass index (weight [kg]/height [m^2^]) estimates were calculated from self-reported weight and height. Underweight and normal weight: <25.0, overweight: 25.0–29.9, and has obesity: ≥30.

^§§^ Needing walking assistance was defined as being unable or finding it very difficult “to walk one-quarter mile without special equipment.”

^¶¶^ Meeting the 2008 aerobic physical activity guideline was defined as participating in ≥150 minutes of moderate-intensity equivalent aerobic activity per week (light- to moderate-intensity minutes plus two times vigorous-intensity minutes).

Among women, the prevalence of walking demonstrated a significant linear increase from 2005 to 2015, with no significant quadratic trend (Figure) (Table 1). This trend remained when stratified by selected characteristics, with two exceptions: both linear and quadratic trends were significant among women who were overweight or lived in the Midwest. The increase in walking prevalence among women between 2010 and 2015 was significant overall (2.7 percentage points) and among select strata (age 45–64 years, age ≥65 years, non-Hispanic whites, college graduates, residents of the Northeast and South regions, those who were underweight or normal weight, those with obesity, and those not needing walking assistance).

Among men, a significant positive linear and negative quadratic trend in reported walking from 2005 to 2015 was observed overall and for most subgroups, with the increase stalling from 2010 to 2015 (Figure) (Table 2). The change in walking prevalence among men from 2010 to 2015 was not significant overall or when estimates were stratified by selected characteristics, with one exception: among men aged ≥65 years, the prevalence of walking increased by 3.8 percentage points from 2010 to 2015.

## Discussion

The prevalence of reported walking for transportation or leisure among men and women increased between 2005 and 2015; however, for men, the increase stalled between 2010 and 2015. This trend among males is similar to trends for leisure time physical activity, with the reported prevalence of meeting physical activity guidelines increasing steadily from 2008 to 2012 and stalling between 2012 and 2015 (*2*).* However, even given this increase, nearly one third of women and men report that they did not walk for at least 10 minutes in the past week.

Walking is an easy way for most adults to incorporate more physical activity into their daily routines. Women are less likely than men to achieve physical activity levels sufficient to meet guidelines (*2*). However, this study found that walking has become increasingly common among women since 2005, representing a potential opportunity for addressing the gender difference in overall physical activity. Efforts to sustain the observed increase in the percentage of adults who walk could contribute to more adults meeting guidelines, potentially reducing the burden of chronic diseases and premature death associated with low levels of physical activity. For example, communities can create additional opportunities for walking by implementing walking programs tailored to the interests and abilities of specific subgroups of the population (*3*). In addition, policies and practices that improve the safety of communities and promote walkable design can help make walking a convenient option for almost all persons.

For both women and men, walking was least prevalent among blacks and persons with lower educational attainment, groups that have been shown to report lower levels of physical activity compared to their counterparts (*8*). In some cases, differences in walking appear to be widening over time. For example, among men, walking increased at a steady rate among college graduates from 2005 to 2015 (significant linear trend only), but stalled between 2010 and 2015 among those who did not graduate from high school (significant linear and quadratic trends). Low socioeconomic status (SES) and minority neighborhoods are often perceived as less attractive and less safe because of traffic or crime when compared with higher SES and majority white neighborhoods (*9*). Efforts to overcome such environmental barriers to walking in these communities, like policies and practices that improve the safety and quality of community supports for physical activity (e.g., trails and sidewalks), might help to reduce the observed disparities in walking (*3*).

The findings in this report are subject to at least four limitations. First, this analysis relies on self-reported data, and social desirability bias might result in overestimates of walking (*10*). Second, the wording of the question about leisure walking changed slightly between 2005 and 2010; to improve comparability between years, participants in all years who reported that a typical walking period lasted <10 minutes (1,076 respondents) were categorized as nonwalkers. Third, survey response rates could contribute to response bias if nonresponders differed systematically from responders, although weighting procedures should reduce the impact of survey nonresponse. Finally, approximately 6% of respondents were missing walking data each year; the application of sample weights would not be expected to mitigate any potential bias associated with missing data.

The reported prevalence of transportation or leisure walking among women and men increased from 2005 to 2015, although among men, the increase has stalled in recent years. By implementing community and street scale design strategies that encourage pedestrian activity and by supporting walking programs where persons spend their time, communities can improve walkability and make walking a safer and easier option for increasing physical activity (*3*).

**Summary**

**What is already known about this topic?**

Only half of U.S. adults report achieving physical activity levels consistent with published guidelines. Walking is an easy way for most persons to be more physically active. Self-reported walking among adults increased by 6 percentage points from 2005 to 2010, but it is unknown whether this increase has continued.

**What is added by this report?**

The prevalence of self-reported walking among women significantly increased from 2005 to 2015 (2005: 57.4%; 2010: 62.5%; 2015: 65.1%); among men, the prevalence increased overall but stalled between 2010 and 2015 (2005: 54.3%; 2010: 61.8%; 2015: 62.8%). Sociodemographic disparities in walking prevalence exist, with the lowest prevalences among non-Hispanic blacks and persons with a high school education or less. Moreover, differences by education level appear to have widened over time among men, with walking prevalence increasing steadily among college graduates but leveling off among men with lower education levels.

**What are the implications for public health practice?**

To promote walking, streets and communities can be designed so that walking is a safe and convenient option for all persons. Communities can also implement walking programs that are tailored to the interests and abilities of specific population subgroups. Focused approaches to overcome barriers to walking in low socioeconomic status and minority communities, such as policies and practices that improve the safety and quality of community supports for physical activity (e.g. trails and sidewalks), might help reduce the observed disparities in walking.

## Footnotes

* https://www.cdc.gov/physicalactivity/downloads/trends-in-the-prevalence-of-physical-activity.pdf.
